# Education of Hematopoietic Stem Cell Transplant Caregivers in Preparation for Their Role

**DOI:** 10.6004/jadpro.2013.4.6.6

**Published:** 2013-11-01

**Authors:** Lynora J. Metoyer

**Affiliations:** From MD Anderson Cancer Center, Houston, Texas

Developing appropriate coping skills, solving problems, and prioritizing are all attributes of an effective caregiver. It is imperative for the caregiver of a patient undergoing hematopoietic stem cell transplantation (HSCT) to maintain his or her own quality of life—including mental, physical, and emotional health—throughout the transplant process to be able to provide optimal care for the HSCT recipient. Unfortunately, self-care is a factor that caregivers often neglect.

We proposed to provide an individualized, structured educational intervention focusing on quality-of-life issues for the HSCT caregiver. This education is warranted to facilitate positive outcomes for the recipient throughout the transplant trajectory, as increased knowledge and access to tools for self-care will better prepare HSCT caregivers for their role, thereby directly impacting outcomes for the transplant recipient.

To this end, a quality improvement study was developed in which an individualized educational intervention was given to a group of caregivers who were then evaluated regarding their comfort level with their role. Their experience was compared with that of a nonintervention group who had received the customary patient-directed education following HSCT. Both groups were given the same questionnaire, yet the participants in the intervention education group were given three additional questions. These questions sought to determine (1) the effectiveness of the intervention and (2) the point during the transplant trajectory at which it was most beneficial for caregivers to receive the education intervention.

## Background

Over the past several decades, HSCT has evolved from an experimental treatment into a sophisticated, highly technical lifesaving therapy (Rice & Bailey, 2009). An estimated 50,000 to 60,000 transplants are performed annually worldwide, and this number will continue to grow. The increased complexity of this treatment creates incremental challenges in the management of the delivery of quality HSCT care (Rice & Bailey, 2009).

The National Cancer Institute (2011) reported that approximately 117,000 people were diagnosed with some form of hematologic malignancy in 2010 and approximately 43,000 succumbed to their disease. Treatment options for these diseases include chemotherapy and radiation therapy. Allogeneic transplant is another viable option for first-line or failed treatment. Other medical indications for transplantation have widened to include both malignant and nonmalignant diseases. According to the National Marrow Donor Program (NMDP), this has led to an increased demand for this therapy; the number of potential transplant patients is expected to double or triple by 2020 (NMDP, 2010). The technology associated with HSCT itself has improved, making it safer for older, sicker patients as well as for those with comorbidities (Rice & Bailey, 2009).

## Caregiver Challenges

Hematopoietic stem cell transplant caregivers encounter a unique situation. While the disease process encountered by the recipient is considered a chronic illness in one respect, there is the expectation that the recipient will evolve from the transplant process as a cancer survivor. Transplant recipients are living longer due to more sophisticated techniques; thus, transplant caregivers may be involved with the care of recipients well beyond the acute phase of transplant (Given, Sherwood, & Given, 2008). It has been reported in the literature that caregivers in general often experience a lack of preparation, knowledge, skills, and confidence needed to be successful in providing care to those with chronic illnesses (Kurtz, Kurtz, Given, & Given, 2005). The transplant caregiver is no exception. It is of the utmost importance for HSCT caregivers to be properly educated and to be given adequate coping skills to maintain their own quality of life in order to provide adequate care to the recipient. The HSCT team should be cognizant of the learning needs and the commitment of the caregiver and provide appropriate support.

Implementation of an evidence-based practice (EBP) individualized educational intervention for HSCT caregivers could potentially be applied to other chronic illnesses outside of HSCT. This practice change could potentially aid in the evolution of new policies for patients and their caregivers across the health-care spectrum.

## Literature Review

While some informal caregivers transition to their role easily, many do not feel confident in what they are undertaking and could benefit from more detailed information and support. A review of the literature maintains that this problem can be addressed by a plan that encompasses the education, needed skill sets, quality-of-life measures, and concerns of the caregiver.

This EBP change project was initiated by conducting an in-depth review of the current literature. Search engines such as Cumulative Index to Nursing and Allied Health Literature (CINAHL), MEDLINE, PubMed, Cochrane Library, and PsycINFO were utilized. The information obtained from this literature review identified several commonalities among caregivers of patients with chronic illnesses in terms of their experiences and the requirements for their success. Little information specific to HSCT caregivers was identified.

In their feasibility study, Hendrix and Ray (2006) noted that it was beneficial to provide individualized caregiver education focusing on home care and managing the symptoms related to cancer and its treatment prior to discharge. Caregivers who received this method of education stated that it enhanced their knowledge and that they felt more confident in the various aspects of their role. The participants believed that the teaching format enhanced their confidence to carry out the caregiver role. Caregiver training required participants to interact, participate in problem-solving exercises for symptom management or other care issues, and perform care-related procedures with the support of an expert.

In a study by Cameron, Shin, Williams, and Stewart (2004), an evaluation of a brief problem-solving intervention for family caregivers of individuals with advanced cancer was performed. Thirty-four caregivers completed a baseline survey and received a brief problem-solving intervention in addition to a detailed home-care guide. The intervention encouraged the caregivers to COPE (be Creative, Optimistic, Plan, and obtain Expert information) to meet the ever-changing challenges associated with the process of caregiving. A telephone follow-up survey performed 4 weeks after the intervention found that the caregivers experienced a decrease in emotional tension as well as an increase in caregiving confidence and problem-solving abilities.

In another descriptive, cross-sectional study by Tamayo, Broxson, Munsell, and Cohen (2010), 194 caregivers of outpatients with leukemia were evaluated to identify strategies geared toward maintaining caregiver quality of life and well-being. Caregiver burden was the most important concern identified, with reference to quality of life, proper administration of medications, and symptom management, as well as psychological factors such as stress and depression. Maintaining positive attitudes, facilitating communication with the health-care team, accessing support, and receiving education were also identified as being important.

## Local Problem

The Department of Stem Cell Transplantation and Cellular Therapy at the MD Anderson Cancer Center, where the practice intervention described in this article was performed, is one of the largest centers in the world for stem cell transplants, performing over 860 such procedures each year.

At the time of the practice change, there was no class or information specifically designed for the HSCT caregiver. While beneficial for patients, the general group discharge classes did not necessarily prepare caregivers for their role. According to Hudson et al. (2008), the group education session model has proven to be a successful intervention, but the sessions are generally not sufficiently thorough, and there is a lack of evidence-based research into the clinical intervention effectiveness of this model.

## Intended Improvement

In the outpatient transplant setting, a trend was noticed that many of the HSCT caregivers were not adequately prepared for their role, especially when it came to their own quality-of-life issues. Caregivers seemed to be so focused on the patient’s care—performing multiple tasks including physical hands-on care and providing psychological and emotional support, all while maintaining two households (their own and that of the patient)—that there was great potential to become overwhelmed. This topic was discussed at various departmental meetings; the consensus was that the caregivers needed more support from the system. The threshold is low for caregivers to actually read and fully comprehend all of the materials that are given to the recipient at the initiation of the transplant process. As an aside, it was also agreed upon that caregivers should be thoroughly assessed for their ability to take on the role prior to the initiation of the transplant process.

## Study Question

The Iowa Model is an EBP model chosen for this change project because of the structured organizational format that allows for application in various clinical settings. Using the Iowa Model, a problem-focused trigger was identified: in this instance, maintaining quality of life while functioning as a stem cell transplant caregiver. The clinical question for this project was, "Does an individualized education intervention help stem cell transplant caregivers prepare for their role?"

## Planning the Intervention

In the months prior to the initiation of the EBP project, key stakeholders within the organization and department were consulted. Once their approval was secured, the project was submitted to the Institutional Review Board, from which an exempt status was granted. The Quality Improvement Assessment Board subsequently approved the project.

The design of this EBP change project consisted of a control group and an intervention group of allogeneic transplant caregivers currently experiencing the recipients’ transition to the outpatient setting, which could be as early as day 12 after transplant. A short information letter introducing the project coordinator and a description specific to each group’s participation in the project were developed. The agreement to participate would serve as the caregiver’s consent. The day prior to the intervention, the caregiver was contacted either in person or by phone to determine if he or she would be accompanying the recipient to their clinic appointment the following day.

A 70-slide Microsoft PowerPoint teaching presentation was created for the intervention group and presented in a one-on-one interaction format. The slides were designed to address the caregivers’ ability to solve problems, development of skill sets, medication and infection overview, symptom management, and coping skills to avoid caregiver burden and stress.

A questionnaire was developed by the project coordinator to gather information assessing the participants’ level of education and their perception of their knowledge of and preparation for the caregiver role. This was a quantitative questionnaire consisting of 20 questions using a Likert scale, which included a rating of 1 to 5, where 1 = very satisfied, 2 = satisfied, 3 = uncertain, 4 = dissatisfied, and 5 = very dissatisfied. Demographics on the participants, including age, race, gender, and relationship, were also included and reported in percentages.

The inclusion criteria consisted of English-speaking adult caregivers with the ability to engage in meaningful conversation to answer questions and provide information. While some of the caregivers were interchanging, most were the primary caregivers for the length of the transplant course. Exclusion criteria included the caregivers of recipients who were acutely ill. Each participant was identified when a transplant recipient was discharged from the hospital or by other advanced practitioners working in the outpatient setting.

The intervention was performed in a quiet, well-lit, unoccupied treatment room to preserve privacy, minimizing distractions and allowing for maximum concentration during the process. The slide presentation was shown to the participants in the intervention group on a laptop computer.

## Methods of Evaluation and Analysis

Customary education included a group intervention primarily aimed at conveying what the patient should expect after discharge, reasons to present to the emergency center, and signs and symptoms of infection and graft-vs.-host disease. Caregivers were also invited to attend, but in some instances they were not able to do so. The customary education also involved a visit from a pharmacist to provide in-depth medication information and a visit from an advanced practice nurse intended to reinforce teaching for the recipient and the caregiver and to make sure postdischarge follow-up had been arranged. Very little (if any) information was provided to the caregiver regarding their role, such as the potential for mental and emotional stress and resources to turn to for help. The individualized education intervention group, on the other hand, solely focused on the caregivers and their needs.

**Figure 1:**
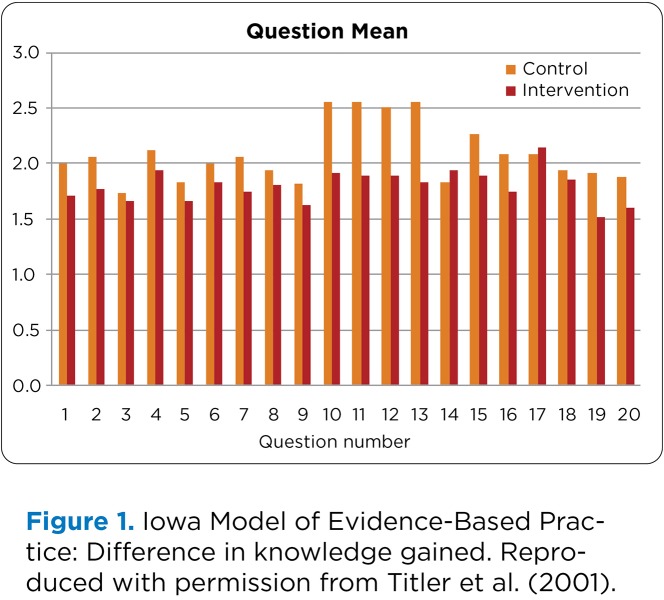
Figure 1. Iowa Model of Evidence-Based Practice: Difference in knowledge gained. Reproduced with permission from Titler et al. (2001).

Questionnaire responses for both groups of caregivers were compared to assess differences between the knowledge gained through the one-on-one education intervention and that acquired from the customary education. The results were reported as the mean and median of each group independently. A mean greater than 2.1 was considered an area of concern (see Figure 1). There was minimal variability during the implementation phase.

## Outcomes

Demographically, the two groups were comparable. An age comparison of both groups revealed that 50% of caregivers were 50 or older. Wives acting as caregivers averaged 50%. More than 80% of the caregivers were Caucasian. Caregivers were more likely to be women. Another significant aspect to report was that 90% of the recipients and caregivers did not reside in the general vicinity of the treatment center. Therefore, they were living in hotels, rented apartments, or trailer/RV parks. While this may not seem important, it is indeed significant when caregiver burden and stress are concerned.

A convenience sample of allogeneic HSCT caregivers in an outpatient ambulatory treatment center was recruited for the project. Seventy potential participants were approached for inclusion. As all 70 individuals agreed to participate, each group had an equal number of subjects (n = 35). The overwhelming desire of the caregivers to participate in the study suggested that there truly was a need for more focused education in the preparation for the caregiver role. Even the caregivers who did not receive the intervention had questions regarding their ability to be an adequate caregiver and how to handle all of what was expected of them without becoming burdened and overstressed.

The project was initiated with a control group participant. Every other participant was given the intervention. Each participant was assigned a number, thus preserving the confidentiality of both the transplant recipient and the caregiver. The slide presentation given to the intervention group took on average 2 hours, allowing time for discussion and questions. After 48 hours, the intervention group was given the 20-question assessment with the addition of 3 questions to determine whether the intervention was beneficial. The questionnaire was read to each group participant in order to facilitate complete responses and provide examples or interpret the meaning of the questions.

**Figure 2:**
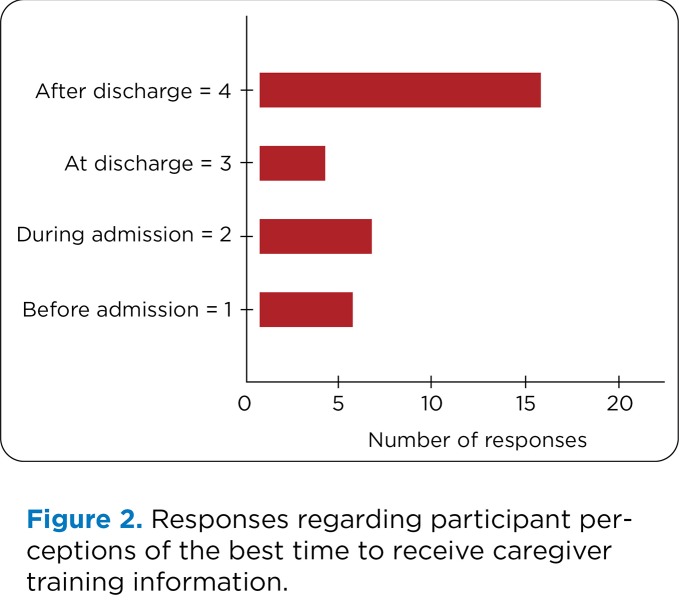
Figure 2. Responses regarding participant perceptions of the best time to receive caregiver training information.

The results yielded a 100% response that the intervention information was needed and beneficial. This group was also given the opportunity to give their opinion on the best timing for the intervention with respect to the transplant process: before admission, during admission, at discharge, or after discharge. The results are noted in a bar graph (see Figure 2). The majority of caregivers felt that the most appropriate timing of the intervention was after discharge, once they have had the chance to experience the outpatient setting. Most felt that this was the most beneficial time because it gave them the opportunity to ask relevant, practical questions about their role as the transition to the outpatient setting occurred.

The control group was asked the same 20 questions, allowing adequate time for discussion and questions. Most members of the control group were satisfied with the customary education and information they received to prepare them for taking care of the patient. However, some of the participants in the control group were interested in receiving more information pertaining to methods to avoid caregiver stress and burden and ways to aid the recipient with psychological and emotional support. Some of the topics covered in the intervention did indeed include strategies for avoiding both emotional and mental caregiver stress. Other concerns expressed by the control group participants included performing tasks and skills adequately, making appropriate decisions, and managing business affairs.

## Study Summary

As identified in the evaluation of this project, some caregivers are not adequately prepared for issues related to the emotional and psychological aspects of their role. In the future, more education in these areas may be beneficial to avoid caregiver burden and stress. The data collected during this practice change project were uniform with respect to the results found in the literature. It is imperative that clinicians identify the caregiver’s learning style, assess his or her readiness to learn, and address any barriers that may hinder assimilation of knowledge.

## Limitations of the Project

The most apparent limitation of the project was the time frame in which the data collection was obtained. The second limitation was the small number of subjects who were interviewed and educated despite the fact that all caregivers who were approached participated. The project coordinator was the only person collecting data. The original goal of 100 participants was not met due to a decreased number of recipient discharges to the outpatient setting. If the project were to be replicated, an increased time period should be considered and a second advanced practitioner would be beneficial in data collection, provided that each educator used the same slide presentation and employed similar teaching styles.

## Interpretations

As the project came to fruition and the data were collected, several ideas and recommendations were identified, including the need for further measures for a practice change. Although this project employed a small sample size, it can be extrapolated to a general consensus that this topic is appropriate for further review. Perhaps this project could be considered the pilot study to support the need for a change in practice to be applied on a larger scale over a longer period of time. Providing a more in-depth individualized approach to the education of transplant caregivers, and potentially caregivers of others with chronic illnesses, may prove beneficial.

## Conclusion

Working through the intricate and varied details of being a transplant caregiver is not an easy feat. Learning new terminology and how it applies to the situation at hand can become overwhelming. After discharge, there is no longer a professional readily available to help make decisions; thus, the onus is placed on the caregiver. Problem-solving becomes an important aspect of the caretaking process. Making weighty decisions on what to do and when to do it is left up to the discretion of the caregiver. The educational presentation included suggestions on making important decisions, solving problems, learning how to cope with an array of possible emotions, and managing to remain physically and emotionally healthy during the process.

The identified "trigger" prompted further investigation into the need for a more focused and individualized approach to the education of the HSCT caregiver. The anticipated outcome of this project was for all caregivers to be equipped with the necessary education, knowledge, and self-help information needed for success in their caregiver role. It is an expectation that this project and future projects with this intent will add to the knowledge in the body of the hematology/oncology nursing discipline and other disciplines as well.
